# Study protocol for First Dental Steps Intervention: feasibility study of a health visitor led infant oral health improvement programme

**DOI:** 10.1186/s40814-022-01195-w

**Published:** 2022-12-03

**Authors:** Patricia N. Albers, Joanna G. Williams, Sarab El-Yousfi, Zoe Marshman, Reena Patel, Rebecca Kandiyali, Katie Breheny, Frank de Vocht, Chris Metcalfe, Robert Witton, Ruth Kipping

**Affiliations:** 1 Bristol Medical School, University of Bristol, Bristol, England; 2 School of Clinical Dentistry, University of Sheffield, Sheffield, England; 3 Healthcare Public Health Directorate, NHS England and NHS Improvement South West, Bristol, England; 4 Centre for Health Economics (CHEW), Warwick Medical School, University of Warwick, Coventry, England; 5 NIHR ARC West, Bristol, England; 6 Faculty of Health: Medicine, Dentistry and Human Sciences, University of Plymouth, Plymouth, England

**Keywords:** Oral health, Dental caries, Health visiting, Public health nursing

## Abstract

**Background:**

Dental caries in childhood is a burden on the daily lives of children and their families, and associated with poor oral health in adulthood. In England, dental caries is the most common reason for young children to be admitted to hospital. It is believed that most tooth extractions (due to decay) for children aged 10 years and under, could be avoided with improved prevention and early management. National public health policy recommendations in England include specific oral health initiatives to tackle tooth decay. One of these initiatives is delivered as part of the Healthy Child Programme and includes providing workforce training in oral health, integrating oral health advice into home visits, and the timely provision of fluoride toothpaste. This protocol seeks to assess the delivery of the First Dental Steps intervention and uncertainties related to the acceptability, recruitment, and retention of participants.

**Methods:**

This study seeks to explore the feasibility and acceptability of the First Dental Steps intervention and research methods. First Dental Steps intervention will be delivered in local authority areas in South West England and includes oral health training for health visitors (or community nursery nurses) working with 0–5-year-olds and their families. Further, for vulnerable families, integrating oral health advice and the provision of an oral health pack (including a free flow cup, an age appropriate toothbrush, and 1450 ppm fluoride toothpaste) during a mandated check by a health visitor. In this study five local authority areas will receive the intervention. Interviews with parents receiving the intervention and health visitors delivering the intervention will be undertaken, along with a range of additional interviews with stakeholders from both intervention and comparison sites (four additional local authority areas).

**Discussion:**

This protocol was written after the start of the COVID-19 pandemic, as a result, some of the original methods were adjusted specifically to account for disruptions caused by the pandemic. Results of this study will primarily provide evidence on the acceptability and feasibility of both the First Dental Steps intervention and the research methods from the perspective of both families and stakeholders.

**Supplementary Information:**

The online version contains supplementary material available at 10.1186/s40814-022-01195-w.

## Background

Dental caries is the most pervasive non-communicable disease, presenting a major global public health challenge, and it is also largely preventable [1]. Dental caries can impact quality of life by disrupting sleep and eating, causing pain, and chronic infections, and causing absenteeism from work or school [1].

Dental caries is a burden on the daily lives of children and their families, with dental caries in childhood predicting poor oral health into adulthood [2, 3]. In England, dental caries is the primary reason for young children to be admitted to hospital. This is not only distressing and disruptive for children and families [4, 5], but also a financial burden on the National Health Service (NHS). In 2020, 35,190 children (0–19 years) were admitted to hospital for the extraction of decayed teeth with an estimated cost of £33 million [6]. It is believed that most tooth extractions (due to decay), for children aged 10 years and under, could be avoided with improved prevention and early management of the dental caries [7]. Findings from Public Health England’s 2019 national dental epidemiological survey of 5-year-old children showed how overall, 23.4% of 5-year-old children in England had experience of obvious dental decay [8]. Furthermore, relative inequalities in the prevalence of dental decay in 5-year-old children have increased from 2008 to 2019 [9].

In England, major policy changes have occurred over the last decade with Local Authorities and the Healthy Child Programme assuming responsibility for oral health improvement [2]. Health Visitor teams and other early years teams are responsible for implementing the healthy child programme. Health visitors are specialist community public health nurses who work with families to support the health and development of infants and children until they are 5 years old. Despite these changes, to date, there is a paucity of evaluations of the impact of oral health improvement initiatives delivered within the Healthy Child Programme on dental caries in children, or on oral health inequalities.

National guidance [10] recommends specific oral health initiatives to tackle dental caries and improve oral health, one of which is delivered as part of the Healthy Child Programme. As part of the healthy child programme, health visitors visit the families of all children below school going age at five mandated development stages shown in Table 1 below [2]. These guidance were updated in March 2021.

**Table 1:** Mandated and suggested health visitor checks [2]

Check (mandated or suggested)	Brief description
Antenatal visit (mandated)	From 28 weeks of pregnancy, delivering comprehensive and holistic assessment of the expectant parents’ needs.
New baby review (mandated)	New baby review, ideally within 10 to 14 days of the birth date.
6- to 8-week review (mandated)	Assessment of progress from birth to 8 weeks.
3- to 4-month contact (suggested)	Growth and development and other key stages, such as social development and interaction.
6 months contact (suggested)	Growth and development and other key stages, such as speech, language, and communication.
9- to 12-month developmental review (mandated)	Review of health and development and the provision of health promotion advice.
2- to 2½-year developmental review (mandated)	General review of child health, development, and growth.

During the 9- to 12-month mandated check good oral health practices and oral health advice are required to be discussed. This is alongside a range of other topics, such as physical, emotional, and social needs; child development; healthy eating; and safety [11]. This initiative is said to demonstrate significant return on investment. For instance Public Health England modelled available evidence on targeted provision of fluoridated toothpaste and toothbrush by post or health visitor and predicted for each £1 spent the return on investment is £4.89 after 5 years and £7.34 after 10 years [3]. This was based on evidence obtained from a rapid review of the evidence on the cost-effectiveness of interventions to improve the oral health of children under 5. This built on a previous version of this that found no cost effectiveness evidence for the provision of fluoridated toothpaste [12]. The updated review found an additional three studies (based in the USA and Australia) that showed the cost effectiveness of the provision of fluoridated toothpaste [3].

Both Public Health England and National Institute for Health and Care Excellence (NICE) have identified limitations in the evidence to support community based oral health interventions and emphasised the need for further research [13, 14].

The Healthy Child Programme recommends that by 6 months children should be introduced to drinking from a cup and by 1 year of age feeding from a bottle should be discouraged [11]. A systematic review of papers that explored the use of fluoride toothpaste in children under 6 years showed that it is effective in reducing dental caries in primary teeth [15]. The majority of the 17 studies included in this review were from higher risk communities. The authors also noted the limited evidence based on different fluoride concentrations; however, it was evident that toothpastes with concentrations above 500 ppb had a great effect on dental caries [15]. In the UK, the recommendation is for very young children to use at least 1000 ppm [16].

The First Dental Steps intervention, was designed and implemented by Public Health England South West, and funded by NHS England and NHS Improvement, and was embedded into the Healthy Child Programme in the South West of England in 2020, thus enabling oral health improvement to be integrated with other health objectives, such as guidance on nutrition and healthy weight. The introduction of this initiative in the South West of England provided the opportunity to explore how this intervention can be fully evaluated if it was introduced on a larger scale across the country.

The First Dental Steps intervention will be delivered in local authority areas in South West England and includes oral health training for health visitors (or community nursery nurses) working with 0–5 year olds and their families. Further, the targeted approach for vulnerable families, includes integrating oral health advice, the provision of an oral health pack (containing a free flow cup, an age-appropriate toothbrush, and 1450 ppm fluoride toothpaste), and sign posting to community dental services. This will be delivered by a health visitor during the 12-month mandated check.

The aim of this study is to conduct a feasibility study of a targeted health visitor delivered intervention (First Dental Steps) to support parents to increase the frequency of infant tooth brushing, to inform a possible future randomised controlled trial. Six objectives were identified to support this aim, they relate to the First Dental Steps intervention and its acceptability plus recruitment, and retention of study participants with a view to a future randomised controlled trial:

1. To explore the current feasibility of delivering the First Dental Steps intervention including different delivery methods and barriers and facilitators to implementation
2. To explore the current acceptability of the First Dental Steps intervention to health visitors and parents
3. To estimate the likely recruitment, in a future RCT, of parent study participants at baseline and retention at follow-up (based on rates from this feasibility study)
4. To determine if the current study methods are acceptable to health visitors and parents
5. To explore the possible effect of the intervention on tooth brushing
6. To pilot and refine methods and resource use data collection to estimate intervention costs and consequences in a future randomised controlled trial

## Methods and design

### Intervention description

The First Dental Steps intervention includes a universal and targeted element, the targeted element is being assessed in this study. The universal element includes offering oral health training for the health visiting teams involved in 0–5 years, preparing them to provide families with evidence-based advice on oral health diet, oral health, and oral hygiene, when to attend the dentist, and signposting to dental services. The training material is based on the ‘NICE Public Health Guidelines [PH55] Oral Health: local authorities and partners’ [14] as well as the ‘PHE Delivering better oral health: an evidence-based toolkit for prevention’ [16]. The learning objectives and workshop programme are provided in Supplementary file 1. The training was delivered in 2-h sessions which were delivered between January 2019 and January 2020. This training was primarily focussed on health education rather than behaviour change.

The targeted element of the First Dental Steps intervention will be delivered to vulnerable families, receiving their 1-year mandated check. There are three components to this element of the intervention including providing families with evidence based oral health care advice and information and an oral health pack. Further, children in these families that are at increased risk of dental caries, identified by having an older sibling that has had teeth extracted under general anaesthetic, will also be referred to local community dental care services for specialist preventative advice and treatment, where the required pathways are in place. The oral health pack includes a free flow cup, an age-appropriate toothbrush, and 1450 ppm fluoride toothpaste.

The health visitors were not given any materials (printed or demonstration items) to take with them into the home visit. Based on their training the HVs were instructed to construct advice for the parents that covered; encouraging them to start brushing their child’s teeth with fluoride toothpaste, advice around diet, and transitioning from baby-bottles or “sucky” beakers. HVs also provided families with details for local dental services, where possible. All of the above was tailored by the HVs to the individual needs of each family, for example some families may have needed more support around encouragement to brush, while others required more information around diet.

Table 2 presents a detailed breakdown of the First Dental Steps intervention using the TiDIER Guidelines [17]. This table includes a detailed description of the intervention, who will receive and deliver it, along with how, when and how often it will be delivered. It also covers any planned changes to the intervention and if modifications are required during the study and finally also explores fidelity.

**Table 2:** Detailed description of intervention using TiDIER guidelines

Item	***Description***	First Dental Steps intervention
**Name**		First Dental Steps
**Why**	*Describe any rationale, theory, or goal of the elements essential to the intervention*	The primary objective of the First Dental Steps intervention is to improve oral health and the uptake of dental services of young children. This is in support of the larger initiative to reduce the number of children requiring extractions under a general anaesthetic. The primary objective of the First Dental Steps Programme will be achieved by maximising the role of health and social care professionals involved in early years care, e.g. health visiting teams In this intervention health visiting teams will, as part of the one-year development check: 1. Provide families with an oral health pack 2. Provide families with evidence based oral health care advice and information 3. Remind families of the importance of children seeing a dentist by the age of one 4. Where appropriate provide signposting to local community dental services 5. Identify vulnerable children at risk of developing decay, and refer to the community dental services
**What**	*Materials: Describe any physical or informational materials used in the intervention, including those provided to participants or used in intervention delivery or in training of intervention providers. Provide information on where the materials can be accessed (such as online appendix, URL)*	1. Training of health visitor teams: Prior to the delivery of the intervention components at the 12-month mandated check health visiting team members will receive additional training on child oral health, this training can be delivered by any certified provider. The training material is based on the ‘NICE Public Health Guidelines [PH55] Oral Health: local authorities and partners’ [12] as well as the ‘PHE Delivering better oral health: an evidence-based toolkit for prevention’ (17). The training was delivered in 2-h sessions which were delivered between January 2019 and March 2021. 2. Material provided to families: The family will be provided with a pack, which includes a free flow cup, an age-appropriate toothbrush, and 1450 ppm fluoride toothpaste.
	*Procedures: Describe each of the procedures, activities, and/or processes used in the intervention, including any enabling or support activities*	First Dental Steps includes a universal and targeted element, the targeted element is being assessed in this study. Universal element: The universal element includes offering oral health training delivered to health visitors or community nursery nurses preparing the attendees to be “Oral Health Champions”. The training aims to enhance their ability and confidence to provide families with evidence-based oral health advice on diet, oral health, and oral hygiene, when to attend the dentist, and signposting to dental services. Targeted elements: Under the current health visiting model, health visiting services offer services on four levels, with Universal Plus and Universal Partnership Plus, being the final two offered to the more vulnerable families. Universal Plus offers rapid response from the local health visiting team when specific expert help is needed. While Universal Partnership Plus, a level provided for the most vulnerable families, delivers ongoing support from health visiting teams and a range of local services (usually social care and other partners) to address more complex issues over a period of time. The targeted element of the First Dental Steps intervention will be for families receiving the Universal Plus or Universal Partnership Plus tier of the HV service. Oral Health Champion trained health visitors will distribute packs (described above) to these vulnerable families. Where possible they will also, set up a direct referral pathway for children identified as being at high risk of developing tooth decay to the local Community Dental Services where they can access specialist preventative advice and treatment.
**Who provides**	*For each category of intervention provider (such as psychologist, nursing assistant), describe their expertise, background, and any specific training given*	The First Dental Steps intervention will be delivered by health visiting team members who will receive additional training on being an Oral Health Champion (described above).
**How**	*Describe the modes of delivery (such as face to face or by some other mechanism, such as internet or telephone) of the intervention and whether it was provided individually or in a group*	The targeted element of the First Dental Steps intervention will be delivered individually to the vulnerable families by the trained health visiting teams. In some local authorities this will be delivered face-to-face, while in others this will be carried out over video conferencing or telephone call. In these situations, the oral health packs will be delivered to the family either by drop-off from a member of the health visiting team or via a collection point or postage.
**Where**	*Describe the type(s) of location(s) where the intervention occurred, including any necessary infrastructure or relevant features*	For both the universal and targeted elements of First Dental Steps, the intervention will take place as part of the professional’s normal operating, as such, some level of advice or sign posting may occur telephonically or remotely. However, for the targeted element, the First Dental Steps intervention will also occur as part of the health visiting team’s mandated checks with vulnerable families, these checks will likely take place in the family home or in established clinics. No additional infrastructure is required.
**When and how much**	*Describe the number of times the intervention was delivered and over what period of time including the number of sessions, their schedule, and their duration, intensity, or dose*	The health visiting team training workshops will take place prior to the study period. The oral health advice and distribution of oral health packs will take place as part of the 12-month mandated check, during a 5-month period. Each child will receive only one pack during the study period. However, families may be in contact with health visiting teams more than once during the study period and may receive additional advice, information, or signposting during the study period.
**Tailoring**	*If the intervention was planned to be personalised, titrated or adapted, then describe what, why, when, and how*	Nothing planned
**Modifications**	*If the intervention was modified during the course of the study, describe the changes (what, why, when, and how)*	The research team will document this throughout the study and report, as need be, with the results.
**How well**	*Planned: If intervention adherence or fidelity was assessed, describe how and by whom, and if any strategies were used to maintain or improve fidelity, describe them*	Intervention fidelity will be assessed through two primary methods. Recording: As part of their standard operating procedure, health visiting teams record a family appointment on an electronic record sheet. For this study, the health visiting team will be requested to record the delivery of advice and the pack as part of this record. To facilitate this, an add-on template was developed by Public Health England in collaboration with Health Visiting Team Leads. The health visiting team will also complete a pre-agreed upon table (called SPIRIT or Participant flow table (Table 4)) documenting each families movement through the study, these data will be anonymous and returned to the research team. Training observations: Attendance at the training will be monitored through an attendance log.
	*Actual: If intervention adherence or fidelity was assessed, describe the extent to which the intervention was delivered as planned*	The research team will document this throughout the study and report, as need be, with the results.

Fidelity to the intervention will be explored by health visitor records of the visit, intervention, and research component, as well as a log of staff attendance at the training sessions.

**Fig. 1:**
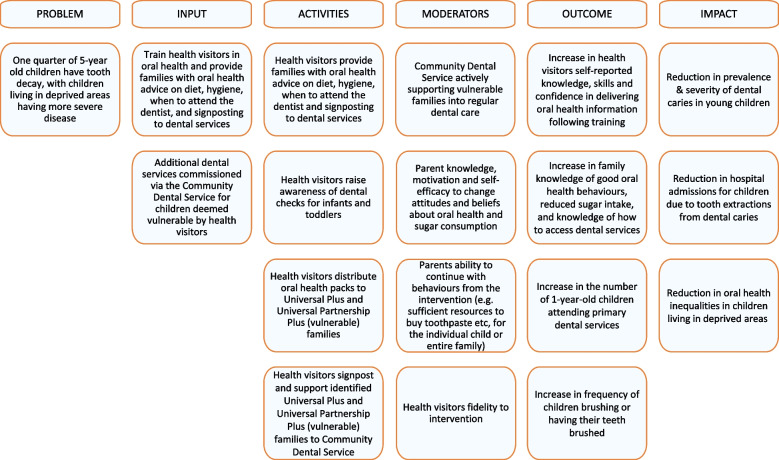
Logic model of the first dental steps intervention

Figure 1 presents a logic model of the First Dental Steps Intervention, demonstrating the public health challenge that the intervention seeks to address or improve, also exploring possible moderators of the intervention, followed by the expected outcomes, and potential impact of the intervention.

### Design and setting

This feasibility study is a non-randomised, pre-post, observation study which follows the 2013 Medical Research Council Guidance for Complex Interventions on conducting a feasibility study prior to conducting a full trial [18], which covers testing procedures, recruitment, and retention.

The study will be based in the South West region of England. The study will be delivered through the health visiting teams which are commissioned at the local authority level. Six local authorities in the South West will be chosen by Public Health England to take part in the intervention. Five of these will be selected for this feasibility study to keep the study manageable within the budget, and also do not have any planned service changes during the study period. Intervention sites will aim to deliver the intervention for 1 month before recruitment begins.

A further four comparison local authorities will be invited to take part. These will be identified from the same region but who were not selected to take part in the First Dental Steps intervention and do not have any other commissioned child oral health interventions at the time. The comparison sites will only be invited to take part in stakeholder interviews, these will be with local authority oral health leads and health visiting team leads.

### Effectiveness outcomes

The effectiveness outcomes for this feasibility study relate to addressing parameters of uncertainty ahead of a larger randomised control trial. The assessment includes, evaluating the feasibility and acceptability of the First Dental Steps intervention, for both families and professionals. Feasibility and acceptability will be assessed using semi-structured qualitative interviews, where prepared topic guides will include specific questions to gauge these, with additional scope for probing by the interviewer. Topics covered will include items such as their experience of the intervention, how well it aligns with the content of the visit, timings (was it the appropriate time for the intervention and was it able to be added to the mandated check), their thoughts on the oral health packs, if the intervention could be improved, and if it should be continued. Recruitment and retention rates of participants and an exploration of the methods relating to this will also be a key outcome. These outcomes form the progression criteria for this feasibility study. A detailed summary of the outcomes, assessment methods, and progression criteria are show in Table 3.

**Table 3:** Summary of the outcomes, assessment methods, and progression criteria

Feasibility:	Assessed via:	Progression criteria:
*Intervention:* was it feasible to implement the First Dental Steps intervention?	a) Interviews with key oral health stakeholders b) Reports on evaluation of training (including results from before and after questionnaires completed by attendees) c) Interviews with health visiting teams about intervention delivery, including training, provision of advice and distribution of oral health packs	• FDS was feasible to implement based on stakeholder and HVT feedback • Training was well received and attended
*Pilot study:* were the study design and methods effective?	a) Recruitment and retention rates b) Were the participant identification and eligibility criteria suitable? c) Were the assessment measures effective: • Complete and missing responses • Participants understanding of questions • Suitability of all variables for the analysis • Any gaps in the data d) Retention of health visiting teams to the study e) Interviews with health visiting teams members f) Interviews with parents g) Further challenges with the study (changes, pauses, or termination) h) Unforeseen limitations or biases	• At least a 30% parental consent rate for eligible children • No more than 30% parental loss to follow-up • Were the participant identification and eligibility criteria suitable? • Were the assessment measures effective: • Complete and missing responses • Participants understanding of questions • Retention of HVTs to the trial
**Acceptability of the intervention:** **Assessed via:**		
Was the intervention acceptable to health visitors and key stakeholders?	a) Interviews with health visiting teams members, health visiting team leads, and local authority leads for oral health	FDS was acceptable to stakeholders, HVTs, and parents, based on interview feedback.
Was the intervention acceptable to parents?	a) Interviews with parents	

### Secondary outcomes

These secondary outcomes are the anticipated primary outcomes for a future randomised controlled trial of the First Dental Steps intervention. These outcomes include oral health behaviours such as tooth brushing frequency, toothpaste use, diet (specifically sugar consumption), and self-reported dental check-ups. In this study, these oral health behaviours will be collected at two time points, using a pre-structured, self-complete questionnaire completed during the 12-month mandated check (baseline) and approximately 5 months later (follow-up).

An economic evaluation will not be conducted in this study. However, we will explore the opportunities and methods for resource use (healthcare staff time, facilities, or consumables) data collection in order to estimate intervention costs and consequences, in a future trial.

### Participants

Vulnerable families (within the intervention local authority areas) will be identified and invited to participate in the intervention and the feasibility study by the health visiting team. As a result of resource constraints study documents will not be translated into different languages, however, as health visitors will be recruiting the families and helping the families complete the consent form and questionnaire, non-English speaking families may be included if the health visitor is able to communicate with them. For the purposes of this study, we define vulnerable families as those who have been identified as needing additional support from the health visiting team (Universal Plus), or multiagency support (Universal Partnership Plus). Only families that provide consent to be involved in the research will be included. As part of the research, we will also invite families to participate in a telephone interview. This interview is an additional step and families can decline to participate. The family can still however complete the baseline and follow-up survey, thus remaining involved in the study. No other inclusion or exclusion criteria will be applied to the recruitment of families.

The study population will be families that meet the inclusion criteria and all eligible families will be invited to participate by the health visiting teams. In total, the five health visiting teams estimated that they may see as many as 789 families during the 6-month study period (01 June 2021 to 31 January 2022).

Qualitative interviews will be undertaken with all agreeing key stakeholders (Local Authority Oral Health Leads and Health Visiting Team Leads) across all the included intervention and comparison local authority areas. Additionally, in the intervention sites, the health visitors who received the training and were involved in the delivery of the intervention and research will also be invited to participate in an interview. We will also invite consenting families to participate in an interview, additional consent will be obtained for these interviews at the time that the family completes the initial consent. At the start of the interview, participants will be asked to verbally confirm their consent. No further inclusion or exclusion criteria will be applied.

A purposive sample of health visiting team members, working in the five intervention local authority areas will be interviewed. A further, purposive sample of key stakeholders including the providers of the oral health training, health visiting team leads, and local authority leads for oral health will also be interviewed. These samples will be selected to ensure representativeness across the different local authorities, health visiting teams, gender, and differing levels of experience or leadership.

A purposively selected sample of parents, who provide consent for the interview will be contacted. The sample will be drawn from the different sites, gender, and ethnic groups to ensure representativeness.

Recruitment for all these interviews will continue until data saturation is reached but it is anticipated that 15–20 interviews will be required for each group (health visiting team members, stakeholders, and parents).

### Public involvement and engagement

A dedicated public involvement and engagement group, including primarily parents of young children will be consulted in relation to the usability and understandability of both the baseline and follow-up questionnaires. This group will also be consulted on the interview topic guide and resource use questionnaire.

### Process

Eligible families will be invited to participate at the point of their 12-month mandated check by the health visiting team. The family will be informed of the steps involved in the research, including the initial questionnaire (baseline), the follow-up questionnaire and an optional telephone interview. Participation is completely voluntary and participants can withdraw at any point without giving a reason. If they are interested in participating in the study the health visiting team will either assist the family with completing a digital or paper-based consent form and the subsequent baseline questionnaire or provide the family with a weblink to complete these independently at a later date. All vulnerable families within the participating local authorities will be provided with the intervention as part of their mandated check. Families provide consent to participate in the research only. Recruitment will take place over a period of 5 months. All participants will be given a copy of their signed consent form, and this will either be a downloadable electronic copy or a paper copy.

**Fig. 2:**
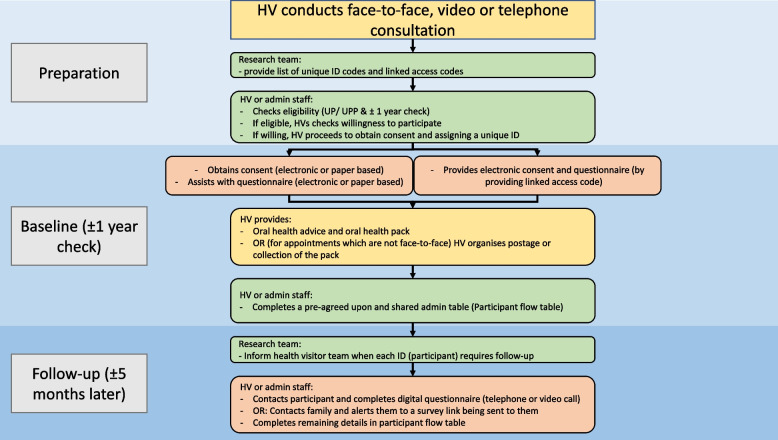
Study procedure overview

For the intervention, data will be collected at two time points; first at the 12-month mandated check (referred to as baseline), and at follow up, approximately 5 months later. A study overview is shown in Fig. 2.

Corresponding to this is a detailed checklist tracking the participants’ movement through the study known as the SPIRIT checklist [19] or participant flow table. This table will be completed by the health visiting team throughout the course of the study. This is shown in Table 4.

**Table 4:** SPIRIT or participant flow table, mapping each respondent’s progress through the intervention and study

Unique ID	Unique ID (from research team)		Code 1
**Survey access code**	**Consent and baseline survey access code**		Access code 1
**Family**	**Siblings had teeth removed under GA**	*Yes/no*	
	**Universal partnership/universal partnership plus classification**	*UP/UPP*	
	**Reason for classification**	*If you do not feel it is appropriate to share please write that here too*	
**Oral health pack**	**Given**	*Yes/no*	
	**Method**	*Home visit/clinic/posted*	
	**Accepted**	*Yes/no*	
	**If not, reason**		
**Consent**	**Participant gave consent**	*Yes/no*	
	**Signed consent electronically or on paper**	*Electronic/paper*	
**Baseline questionnaire**	**Completed questionnaire**	*Yes/no*	
	**Electronic or paper**	*Electronic/paper*	
	**Date completed**	*dd/mm/yyyy*	
	**Time**	*(if possible or rough time, AM or PM)*	
	**For paper based: family/ health visitor will post consent and questionnaire**	*Family/HV*	
**Expected follow-up date**	**Expected follow-up date**	*dd/mm/yyyy*	
**Follow-up questionnaire**	**First contact**	*Reached/did not reach*	
	**Second contact**	*Reached/did not reach*	
	**Third contact**	*Reached/did not reach*	
	**Completed on call**	*Yes/no*	
	**Completion date**	*dd/mm/yyyy*	
	**Link sent**	*Yes/ No*	

### Data collection and instruments

Baseline and follow-up questionnaire data will be collected using a pre-structured, self-completed digital questionnaire, completed with the assistance of the health visiting team members where possible. The questionnaire is based on three existing validated measures and was tailored for this study with support of a patient and public involvement group. The National Children's Dental Health Survey, 2013 (parent and child questionnaires) [20], these questionnaires were developed by stakeholder consultation, and they underwent expert review, cognitive testing, pilot testing, and ethical review [21]. Additionally, the Born in Bradford: 12 months questionnaire [22], a questionnaire originally based on validated measures and any bespoke questions were tested within their sample and modified accordingly [23]. Finally, the Strong Teeth study questionnaire [24], a questionnaire based on validated measures, was also consulted [24]. We then tailored our questionnaire with support of a patient and public involvement group. The questionnaire is two pages long, with nine questions, and should take no more than 10 min to complete. The key areas that the questionnaire covers are current tooth brushing frequency, diet, and dental check-ups. Demographic details such as the age of the child, relationship of the parent, guardian, or carer of the child that is completing the questionnaire, socio-economic status, ethnicity, and if the child has any older siblings are also included. Our questionnaire was developed to include items from existing measures, however, there is limited data on the reliability of the use of self-report oral health or hygiene practices, with some previous research exploring this among adolescents in Brazil [25].

These questionnaires will be delivered online via REDCap, a secure online data capture system hosted by the University of Bristol. As the research team are unable to view any family details, unique ID codes that correspond to unique access codes which link to the consent forms and baseline questionnaire will be provided to the health visiting team. After completing the consent forms the respondent will be automatically directed to the baseline questionnaire. Linked forms to capture participant details for the interviews and ‘thank you’ vouchers will also be created in the REDCap system. For families that complete both of the two questionnaires, we will offer a £10 *Love to Shop* shopping voucher. This will be an e-voucher that will be emailed to participants on receipt of the second questionnaire. We will collect their email address, or a postal address if an email address is not available, at the end of the follow-up questionnaire.

The interviews with parents who received the intervention will be conducted shortly after completing the baseline survey and will also be conducted via telephone and recorded. These interviews will explore the acceptability of the intervention in terms of the information and signposting provided by the health visiting team as well as the packs, the timing of the intervention and the study methods. Additionally, barriers and facilitators to applying any learning from the intervention, presently and in the future, will also be explored. We will offer these parents an additional £10 *Love to Shop* shopping voucher. Similarly, this will be an e-voucher that will be emailed to participants. We will collect their email address at the end of the interview or a postal address if an email address is not available.

The interviews with health visiting team member and stakeholders will also be conducted via telephone or via online platforms, such as Google Meet and Microsoft Teams, and audio-recorded. These will explore the acceptability of the intervention from their perspective within their different roles. During the interview, topics such as training, the practicality of including the intervention into the mandated check, how well the content of the packs were received, the timing of the intervention, the study methods, and the possibility of future implementation will be explored. Further, we will explore their views on randomising health visiting teams or local authorities to the intervention.

### Data management

Questionnaire data will be collected predominantly digitally using a secure online data entry and management system (REDCap). Any paper-based questionnaire data will be entered into the same system by a researcher. After the consent and questionnaires have been submitted only the research team will have access to these data, however, at the time of submission the family will have the option to save a pdf copy of their signed consent form. In the event of paper copies being used, these will be sent to the research team to enter into the digital system. All questionnaire data will be collected and stored in anonymised form using unique participant identification numbers. Participant identification numbers and corresponding participant names will be held by the health visiting teams only. All personal data, such as first name and contact details, that are collected by the researchers, with the consent of the participants, will be stored in separate files from the questionnaire data. These data will be stored on a secure server and only members of the research team will be able to access it; these data will be destroyed after use.

All interviews will be audio-recorded and transcribed verbatim by a transcription service approved by the research sponsor, no participant names will be used in the transcriptions. A list of participant names and their unique identification number will be held in a separate location. Digital recordings of interviews will be stored securely and will be held separately from transcripts and information on participant identities. In reporting the results, care will be taken to use quotations which do not reveal the identity of respondents and anonymised data will be used wherever possible.

Participant details will be anonymised in any publications that result from the study. Personal data (e.g. name and address, or any data from which a participant might be identified) will not be kept for longer than is required for the purpose for which it has been acquired. Anonymised research data will be kept for no longer than 5 years in accordance with the requirements of the research sponsor as well as the Caldicott Principles, UK Data Protection Act 2018 and GDPR.

### Economic evaluation

The costs of the intervention will be gathered, these costs will include the cost of the oral health packs, the cost of the commissioned training workshops, and additional training costs incurred by health visiting team, such as staff time and travel. Staff time will be estimated based on the number of staff members attending the training and the length of the training. We will also endeavour to explore additional travel costs for the teams in travelling to the training workshops that were held face-to-face. Additionally, we will explore with parents, during the telephone interviews, if they incurred any additional costs as a result of wanting to continue with the behaviours from the intervention, either for that child or for the entire family.

### Analysis

Quantitative analysis of consent, recruitment rates, and response rates of parents will be descriptive (means and standard deviation, or number and percentage) as appropriate. Descriptive comparisons of these data will be made between local authorities. Loss to follow-up and missing data will also be reported. These descriptive analyses will address objective 3. Additionally, descriptive summaries of all the demographic details will be provided.

Qualitative data will be analysed using framework analysis [26] as it provides a pragmatic approach which produces results that can be easily incorporated into mixed-method studies [27]. A deductive-inductive hybrid approach will be adopted allowing for the exploration of the research questions and a *priori* themes, identified from the literature search, while also allowing for new themes to be identified. The analysis will involve the following stages: identifying initial themes, labelling the data, sorting the data by theme, and synthesising the data.

For the economic analysis, in accordance with objective 6, evaluation of resource use and costs will be limited to descriptive statistics, leading to refined methods for a future randomised controlled trial.

## Discussion

This protocol was written after the start of the COVID-19 pandemic. In consultation with the participating health visiting teams, we adjusted some of the methods to account for disruptions caused by the pandemic. Some of the changes included the provision for mandated checks to be held remotely. Additionally, while during initial planning we were offering a mix of paper options for the questionnaires, with the pandemic, the digital option was preferred.

Although the First Dental Steps intervention is directed at young children and their families, we will not be collecting any direct data from the young children. Parents or guardians (with the help of the health visiting team) will be asked to complete two short questionnaires. Further, there are no risks to the families or children as a result of using any items in the oral heath pack.

Results of this study will primarily provide evidence on the acceptability and feasibility of both the First Dental Steps intervention and the research methods. This will be from the perspective of not only the families but also a range of stakeholders such as the health visiting teams involved with the delivery. Findings about the acceptability and feasibility of the intervention will be fed back to the intervention funder. Findings of the study will inform decisions about and the design of a potential future randomised controlled trial of First Dental Steps intervention.

### Study limitations

We have identified two primary limitations to our study, firstly the lack of randomisation, as specific local authorities in the South West of England were selected by Public Health England to implement the intervention. We therefore do not understand the challenges around recruiting and randomising local authority areas. However, we will explore this in the qualitative interviews with stakeholders. Secondly, in our study, we are not able to explore long term outcomes such as the ‘decayed, missing, or filled teeth’ (dmft) index, which ideally would be follow-up at age 5, this is due to funding. As such, we are not able to explore the participant retention over this period of time nor the ability to collect this outcome data.

## Supplementary Information

**Additional file 1: Supplementary file 1.** HV Team training.

## Data Availability

Not applicable.

## Abbreviations

dmft: Decayed missing or filled teeth
NHS: National Health Service

## References

[1] World Health Organization. Sugars and dental caries. [Internet]. World Health Organization. Published 2017. Available from: https://apps.who.int/iris/bitstream/handle/10665/259413/WHO-NMH-NHD-17.12-eng.pdf. [Accessed: 04/08/2022].

[2] Public Health England, PHE. Healthy child programme 0 to 19. [Internet]. London. Published 20 January 2016, Updated 17 March 2021. Available from: https://www.gov.uk/government/publications/healthy-child-programme-0-to-19-health-visitor-and-school-nurse-commissioning. [Accessed: 24/01/2022].

[3] Public Health England, PHE. A rapid review of evidence on the cost-effectiveness of interventions to improve the oral health of children aged 0-5 years. [Internet]. PHE publications gateway number: 2016321. Published September 2016. Available from: https://assets.publishing.service.gov.uk/government/uploads/system/uploads/attachment_data/file/560972/Rapid_review_ROI_oral_health_5_year_old.pdf. [Accessed: 15/06/2020].

[4] Rodd HD, Hall M, Deery C, Gilchrist F, Gibson B, Marshman Z (2013). Video diaries to capture children’s participation in the dental GA pathway. Eur Arch Paediatr Dent.

[5] Levine RS (2021). Childhood caries and hospital admissions in England: a reflection on preventive strategies. Br Dent J.

[6] Public Health England, PHE. Research and analysis. Hospital tooth extractions of 0 to 19 year olds. Episodes of children being admitted to hospital for tooth extractions from 2016 to 2020. [Internet]. Published: 6 March 2019. Available from: https://www.gov.uk/government/publications/hospital-tooth-extractions-of-0-to-19-year-olds. [Accessed: 04/08/2022].

[7] Publication, Part of NHS Outcomes Framework (NHS OF) NHS Outcomes Framework Indicators – December 2020 Supplementary Release. Published 17/12/2020. Available from: https://digital.nhs.uk/data-and-information/publications/statistical/nhs-outcomes-framework/december-2020-supplementary-release. [Accessed: 28/01/2021].

[8] Public Health England, PHE. National Dental Epidemiology Programme for England: oral health survey of 5-year-olds 2019. [Internet]. London. Published March 2022. Available from: https://assets.publishing.service.gov.uk/government/uploads/system/uploads/attachment_data/file/873492/NDEP_for_England_OH_Survey_5yr_2019_v1.0.pdf. [Accessed: 24/01/2022].

[9] Public Health England, PHE. Inequalities in oral health in England. [Internet]. London. Published March 2021. Available from: https://www.gov.uk/government/publications/inequalities-in-oral-health-in-england. [Accessed: 24/01/2022].

[10] Public Health England, PHE. Local authorities improving oral health: commissioning better oral health for children and young people. An evidence-informed toolkit for local authorities. [Internet]. PHE publications gateway number: 2014147. Published: June 2012. Available from: https://assets.publishing.service.gov.uk/government/uploads/system/uploads/attachment_data/file/321503/CBOHMaindocumentJUNE2014.pdf. [Accessed: 05/08/2022].

[11] Department of Health., Healthy Child Programme Pregnancy and the first five years. [Internet]. PHE gateway number: 12793. Published: 27 October 2009. Available from: https://assets.publishing.service.gov.uk/government/uploads/system/uploads/attachment_data/file/167998/Health_Child_Programme.pdf. [Accessed: 11/01/2022].

[12] Coffin D, Craig JC, Arber M, & Glanville J. Literature review of economic evaluations on oral health improvement programmes and interventions. York: York Health Economics Consortium; 2013. Available from: https://www.nice.org.uk/guidance/ph55/evidence/literature-review-of-economic-evaluations-on-oral-health-improvement-programmes-and-interventions-pdf-6540668317. [Accessed: 04/08/2022].

[13] Public Health England, PHE. Healthy child programme: Rapid Review to Update Evidence. [Internet]. PHE publications gateway number: 2014716. Published: 6 March 2015. Available from: https://www.gov.uk/government/publications/healthy-child-programme-rapid-review-to-update-evidence. [Accessed: 15/06/2020].

[14] National Institute for Health and Care Excellence. Oral health: local authorities and partners. Public health guideline [PH55]. [Internet]. NICE: London. Published: 22 October 2014. Available from: https://www.nice.org.uk/guidance/ph55/resources/oral-health-local-authorities-and-partners-pdf-1996420085701. [Accessed: 15/06/2020].

[15] Wright JT, Hanson N, Ristic H, Whall CW, Estrich CG, Zentz RR (2014). Fluoride toothpaste efficacy and safety in children younger than 6 years: A systematic review. J Am Dent Assoc.

[16] Office for Health Improvement and Disparities, Department of Health and Social Care, NHS England, and NHS Improvement. Delivering better oral health: an evidence-based toolkit for prevention. [Internet]. Published: 12 June 2014, Updated 9 November 2021. Available from: https://www.gov.uk/government/publications/delivering-better-oral-health-an-evidence-based-toolkit-for-prevention. [Accessed: 24/01/2022].

[17] Hoffmann TC, Glasziou PP, Boutron I, Milne R, Perera R, Moher D, et al. Better reporting of interventions: template for intervention description and replication (TIDieR) checklist and guide. BMJ. 2014:348. 10.1136/bmj.g1687. 10.1136/bmj.g1687 24609605

[18] Craig P, Dieppe P, Macintyre S, Michie S, Nazareth I, Petticrew M (2013). Developing and evaluating complex interventions: The new medical research council guidance. Int J Nurs Stud.

[19] Chan AW, Tetzlaff JM, Altman DG, Laupacis A, Gøtzsche PC (2013). SPIRIT 2013 Statement: Defining Standard Protocol Items for Clinical Trials. Ann Intern Med.

[20] NHS Children’s Dental Health Survey. The Children’s Dental Health Survey 2013, England, Wales and Northern Ireland. NHS Digital. Published: 19 March 2015. Available from: https://digital.nhs.uk/data-and-information/publications/statistical/children-s-dental-health-survey/child-dental-health-survey-2013-england-wales-and-northern-ireland#resources. [Accessed: 15/06/2020].

[21] Children’s Dental Health Survey. The Children’s Dental Health Survey 2013, Technical Report: England, Wales and Northern Ireland. NHS Digital. Published: 19 March 2015. Available from: https://files.digital.nhs.uk/publicationimport/pub17xxx/pub17137/cdhs2013-technical-report.pdf. [Accessed: 16/09/2022].

[22] Born in Bradford. The Born in Bradford 12 months questionnaire. [Internet]. Available from: https://borninbradford.nhs.uk/wp-content/uploads/Born-in-Bradford_12months_BiB1000_20160526.pdf. [Accessed: 15/06/2020].

[23] Wright J, Fairley L, McEachan R, et al. Development and evaluation of an intervention for the prevention of childhood obesity in a multiethnic population: the Born in Bradford applied research programme. Southampton (UK): NIHR Journals Library; 2016 May. (Programme Grants for Applied Research, No. 4.6.) Recruitment and data collection for the BiB1000 cohort to investigate childhood obesity. Available from: https://www.ncbi.nlm.nih.gov/books/NBK362167/. [Accessed: 16/09/2022]. 27252997

[24] Tull K, Gray-Burrows KA, Bhatti A (2019). “Strong Teeth”—a study protocol for an early-phase feasibility trial of a complex oral health intervention delivered by dental teams to parents of young children. Pilot Feasibility Study.

[25] Gil GS, Morikava FS, Santin GC (2015). Reliability of self-reported toothbrushing frequency as an indicator for the assessment of oral hygiene in epidemiological research on caries in adolescents: a cross-sectional study. BMC Med Res Methodol.

[26] Ritchie J, Lewis J (2003). Qualitative Research Practice: A Guide for Social Science Students and Researchers.

[27] Gale NK, Heath G, Cameron E, Rashid S, Redwood S (2018). Using the framework method for the analysis of qualitative data in multi-disciplinary health research. BMC Med Res Methodol.

