# Supplementary material for: Study protocol for First Dental Steps Intervention: feasibility study of a health visitor led infant oral health improvement programme
Source: Pilot Feasibility Stud. 2022 Dec 3;8:245. doi: 10.1186/s40814-022-01195-w (PMC9719194; doi:10.1186/s40814-022-01195-w)
Supplement: Supplementary file 1 — Additional file 1: Supplementary file 1. HV Team training. [file 40814_2022_1195_MOESM1_ESM.docx]

Supplementary file 1_ HV Team training

Summary of learning objectives based on NICE recommendations: 7, 8, 9, 12, 13, 14

**Objective:** To ensure that the health visiting team can provide evidence based oral health information and advice incorporated into topics on health, wellbeing, diet, nutrition, and parenting.

This is based on the following themes:

- The fact that tooth decay is preventable, and how fluoride can help prevent it
- How good oral health contributes to children's overall health, wellbeing and development
- The consequences of poor oral health for children
- Causes, symptoms and how to prevent tooth decay
- Promotion of breastfeeding and healthy weaning, including how to move from breast or bottle feeding to using an open cup by 12 months
- The links between dietary habits and tooth decay, and promotion of food, snacks (for example, fresh fruit) and drinks (water and milk) that are part of a healthier diet
- The importance of regular tooth brushing, and promoting the use of fluoride toothpaste as soon as teeth come through
- The links between health inequalities and oral health and the needs of groups at high risk of poor oral health
- Encouraging people to regularly visit the dentist from when a child gets their first tooth
- Encouraging and supporting families to register with a dentist, and where to get advice about local dental services
- Giving a practical demonstration of how to achieve and maintain good oral hygiene and encouraging tooth brushing from an early age
- Using sugar-free medicine

Training workshop programme:

| **Time** | **Activity** | **Detail** | **Resources** |
| --- | --- | --- | --- |
| 10 mins | Introduction | - Welcome, scene setting, background info - Everyone introduces themselves - Explain how the workshop will run - Sign in sheet/ feedback forms to complete at end | - Sign in/contact details - Feedback form |
| 10 mins | What is Dental First Steps? | - Details on why programme is running - Facts and figures on GA extractions | - Power point slides/laptop |
| 20 mins | The importance of tooth brushing | - How can you start the conversation? - What experience/skills can you use to engage the families? - Promoting the family packs: free flow cup in relation to oral health/development | - Open group discussion - Idea sharing, techniques - Example of family pack |
| 15 mins | What is tooth/bottle decay | - Explain the process of decay: different stages - Frequency of sugar: poor brushing – plaque – acid – tooth/ bottle decay | - Laminated photos of tooth decay - Models: bottle caries, stages of decay |
| 15 mins | How can we prevent this? | - Education: key oral health messages - Fluoride: what is ppm - Best practice: Delivering Better Oral Health - Reinforcing healthy eating options: reducing sugar intake | - A4 Top tips flip chart - Selection of toothbrushes and tooth paste with different ppm - Drinks/snacks with sugar content |
| 10 mins | Tooth brushing techniques | - How to encourage brushing? - Demonstration of techniques that can be used for children - Group to practise technique | - Dummy doll for demonstration |
| 10 mins | Conclusion | - Questions and answers - Signpost referral pathway for each specific location - Feedback sheets to be completed | - Open discussion - Contact details - Feedback forms |
